# Polymorphic Structure Determination of the Macrocyclic Drug Paritaprevir by MicroED

**DOI:** 10.1002/adbi.202300570

**Published:** 2024-02-21

**Authors:** Guanhong Bu, Emma Danelius, Lianne H.E. Wieske, Tamir Gonen

**Affiliations:** 1. Department of Biological Chemistry, University of California Los Angeles, 615 Charles E. Young Drive South, Los Angeles, CA 90095, USA.; 2. Howard Hughes Medical Institute, University of California Los Angeles, Los Angeles, CA 90095, USA.; 3. Department of Chemistry – BMC, Uppsala University, Husargatan 3, 75237 Uppsala, Sweden.; 4. Department of Physiology, University of California Los Angeles, 615 Charles E. Young Drive South, Los Angeles, CA 90095, USA.

**Keywords:** MicroED, Macrocycles, Polymorphism, Molecular Chameleons, HCV Protease

## Abstract

Paritaprevir is an orally bioavailable, macrocyclic drug used for treating chronic Hepatitis C virus infection. Its structures had been elusive to the public until recently when one of the crystal forms was solved by MicroED. In this work, we report the MicroED structures of two distinct polymorphic crystal forms of paritaprevir from the same experiment. The different polymorphs show conformational changes in the macrocyclic core, as well as the cyclopropyl sulfonamide and methyl pyrazinamide substituents. Molecular docking shows that one of the conformations fits well into the active site pocket of the NS3/4A serine protease target, and can interact with the pocket and catalytic triad via hydrophobic interactions and hydrogen bonds. These results can provide further insight for optimization of the binding of acyl sulfonamide inhibitors to the NS3/4A serine protease. In addition, this also demonstrates the opportunity to derive different polymorphs and distinct macrocycle conformations from the same experiments using MicroED.

## Graphical Abstract

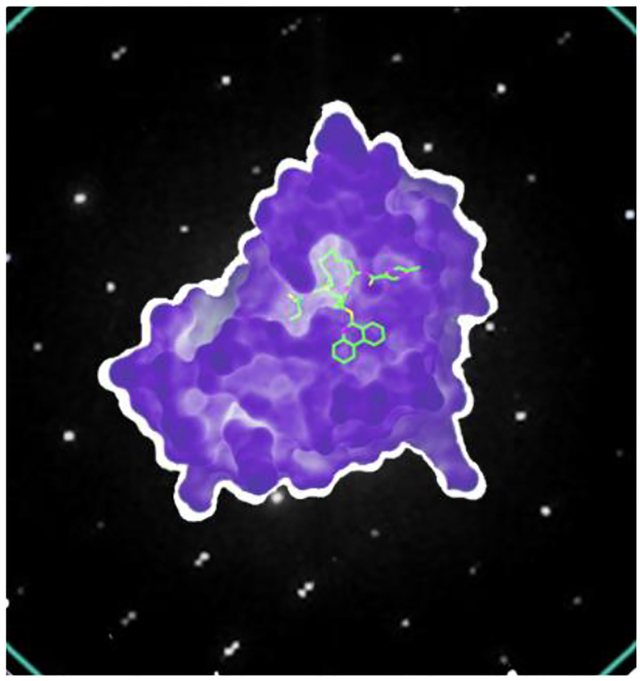

Paritaprevir has been widely used for treating chronic Hepatitis C virus infection. It is a macrocyclic drug whose structure has long been unavailable. Microcrystal electron diffraction is applied to determine two polymorph structures which are investigated for potential binding mode to the target protease.

## Introduction

Polymorphism, the phenomenon where a solid chemical compound occurs in more than one crystalline form with different molecular packing and/or conformation can result in different drug properties such as solubility, stability, bioavailability, potency, and toxicity, and is therefore of high importance to pharmaceutical research and development. [1–3] Active pharmaceutical ingredients (APIs) in pure drugs or formulated products are commonly available to patients as solid forms, thus it is of critical importance to understand and control polymorphism for optimal drug performance. [4, 5] Polymorphism can be characterized by a combination of techniques including X-ray diffraction (single crystal or powder) and spectroscopy (Raman, infrared or NMR), [2] and insufficient characterization occasionally can cause the inactivation of drugs for life-threatening diseases. One infamous example is the anti-AIDS drug ritonavir which was commercialized based on the only previously described crystal form. The discovery of a polymorph in the commercial capsules with reduced solubility and bioavailability led to its temporary withdrawal from the market. [6] Molecular properties such as size and conformational flexibility can strongly influence the propensity of forming crystalline polymorphs, hence the elucidation of polymorphism is of higher importance outside the classical rule of five (Ro5) space for drug development.

**Figure 1.**
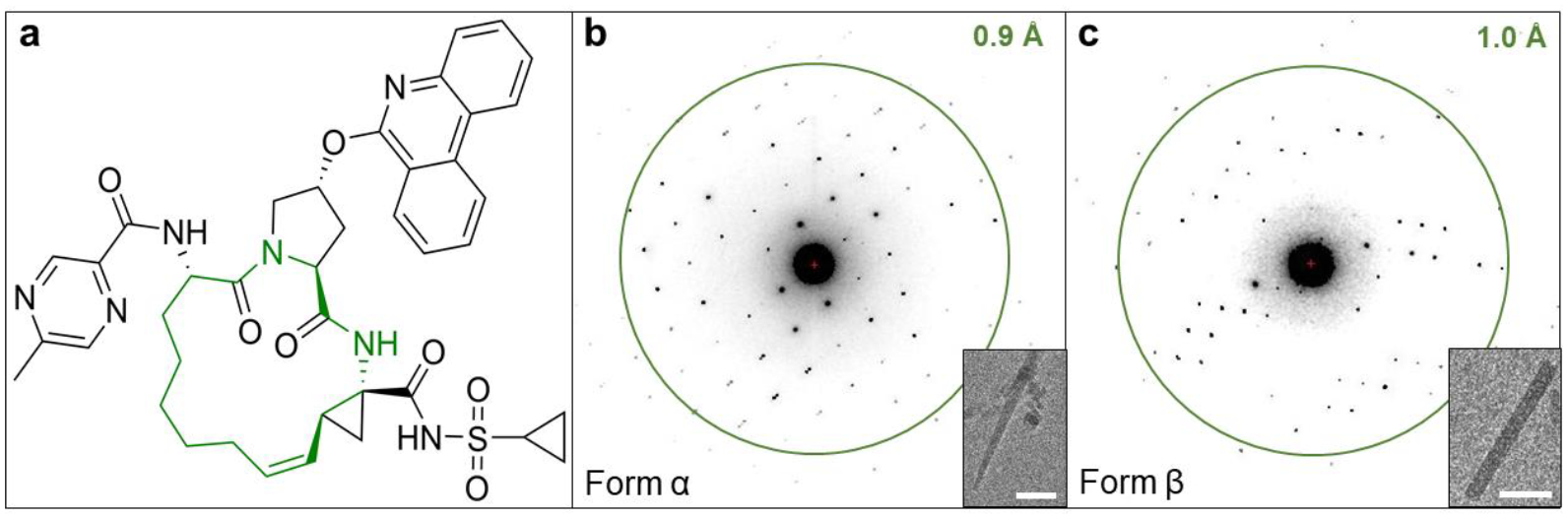
**a:** Chemical structure of paritaprevir with the macrocyclic core highlighted in green. **b:** Representative crystal image of needle-like paritaprevir form α and MicroED pattern. **c:** Representative crystal image of rod-like paritaprevir form β and MicroED pattern. Scale bars correspond to 2 μm.

Macrocycles are defined as having a cyclic core with 12 or more heteroatoms and represent novel chemical modalities beyond the traditional rule of 5 (bRo5) for oral absorption. [7, 8] Compared with classic small molecule drugs within Ro5, macrocycles are more flexible and complex, can adopt multiple conformations and bind to difficult-to-drug protein targets including those with high mutation rates or flat surfaces. [9, 10] Due to the conformational flexibility of macrocyclic drugs, they are prone to be crystallized as polymorphs. [11] Paritaprevir (ABT-450, Figure 1) is one of the highest molecular weight drugs approved for oral administration. [12] It was developed by AbbVie and Enanta Pharmaceuticals as a potent inhibitor of hepatitis C virus (HCV) non-structural 3/4A protease which plays a vital role in viral replication and assembly. [13, 14] Paritaprevir was approved as a component of direct-acting anti-HCV combination therapy under the brand names Viekira Pak (along with ombitasvir, ritonavir and dasabuvir) and Technivie (along with ombitasvir and ritonavir). [14, 15] Despite its wide use in clinics, there are no target-bound structures in the Protein Data Bank (PDB), and only 2 crystal forms can be determined by powder or single crystal X-ray diffraction, [16–18] illustrating the difficulty in studying these large and flexible macrocyclic structures. Using a SerialEM-based high-throughput microcrystal electron diffraction (MicroED) data collection, we recently solved one of the crystal forms of paritaprevir, hereafter referred to as form α. [19] MicroED is a cryogenic electron microscopy (cryo-EM) method for structure determination from tiny crystals of micron to sub-micron size, bypassing the crystallization assay required for conventional X-ray diffraction. [20–22] Recent works have shown the ability of MicroED to solve polymorphic structures of small molecules including glycine by time-resolved in situ crystallization, [23] diketopyrrolopyrroles by drop casting on EM grids, [24] indomethacin by crystallization screening, [25] bis-arylacylhydrazone in the presence or absence of hydrates, [26] and vemurafenib by melt crystallization. [27]

In this work, we report two polymorphic crystal forms of paritaprevir obtained from the same powder preparation and experiment, without any crystallization assay, by high-throughput MicroED using SerialEM. The new crystal structure, herein referred to as paritaprevir form β, shows a different conformation and unique packing pattern, and hence reveals a conformational polymorphism from the same sample. Molecular docking experiments show that only form β can bind to the active site pocket of HCV NS3/4A protease and interact with the catalytic triad, whereas form α doesn’t fit into the pocket. Hence, form β is a potential target-bound conformation.

## Results and discussion

### MicroED data collection and data processing.

The MicroED sample preparation and grid screening for paritaprevir were performed according to previously described protocols. [19, 28] For successful application of microcrystals onto the TEM grids, the powder samples were dried during methanol evaporation in the fume hood. Two major crystal forms were identified from the same grid preparation using low-magnification whole-grid atlases. Paritaprevir form α yielded needle-like microcrystals with dimensions of 4–10 μm in length and 0.1–0.5 μm in width (Figure 1b). Initial data collection confirmed that the space group and unit cell dimensions of form α match our recently published MicroED structure of paritaprevir [19] and paritaprevir hydrate form I. [16, 17] Paritaprevir form β produced rod-like microcrystals (Figure 1c), typically 2–5 μm in lengths and 0.2–0.4 μm in widths. Using automation, MicroED data were recorded on a Thermo-Fisher Falcon III detector at an electron dose rate of 0.01 e^−^ Å^−2^ s^−1^ and 1 second exposure per frame as the sample stage was continuously rotated from −30° to +30° at 1° per second. The data were processed in XDS following previously published procedures. [28] The *ab initio* structure of form α was solved in the orthorhombic space group P2_1_2_1_2_1_ (a = 5.09 Å, b = 15.61 Å, c = 50.78 Å, and α = β = γ = 90°) from a merged dataset with an overall completeness of 89%, and refined at 0.85 Å to an R_1_ value of 0.1472 (Table S1, Figure S1). The *ab initio* structure of form β was solved in the same orthorhombic space group P2_1_2_1_2_1_ but with significantly different unit cell size (a = 10.56 Å, b = 12.32 Å, c = 31.73 Å, and α = β = γ = 90°) from a merged dataset with an overall completeness of 98.4%, and refined at 0.95 Å to an R_1_ value of 0.1347 (Table S1, Figure S3).

### Structure analysis.

**Figure 2.**
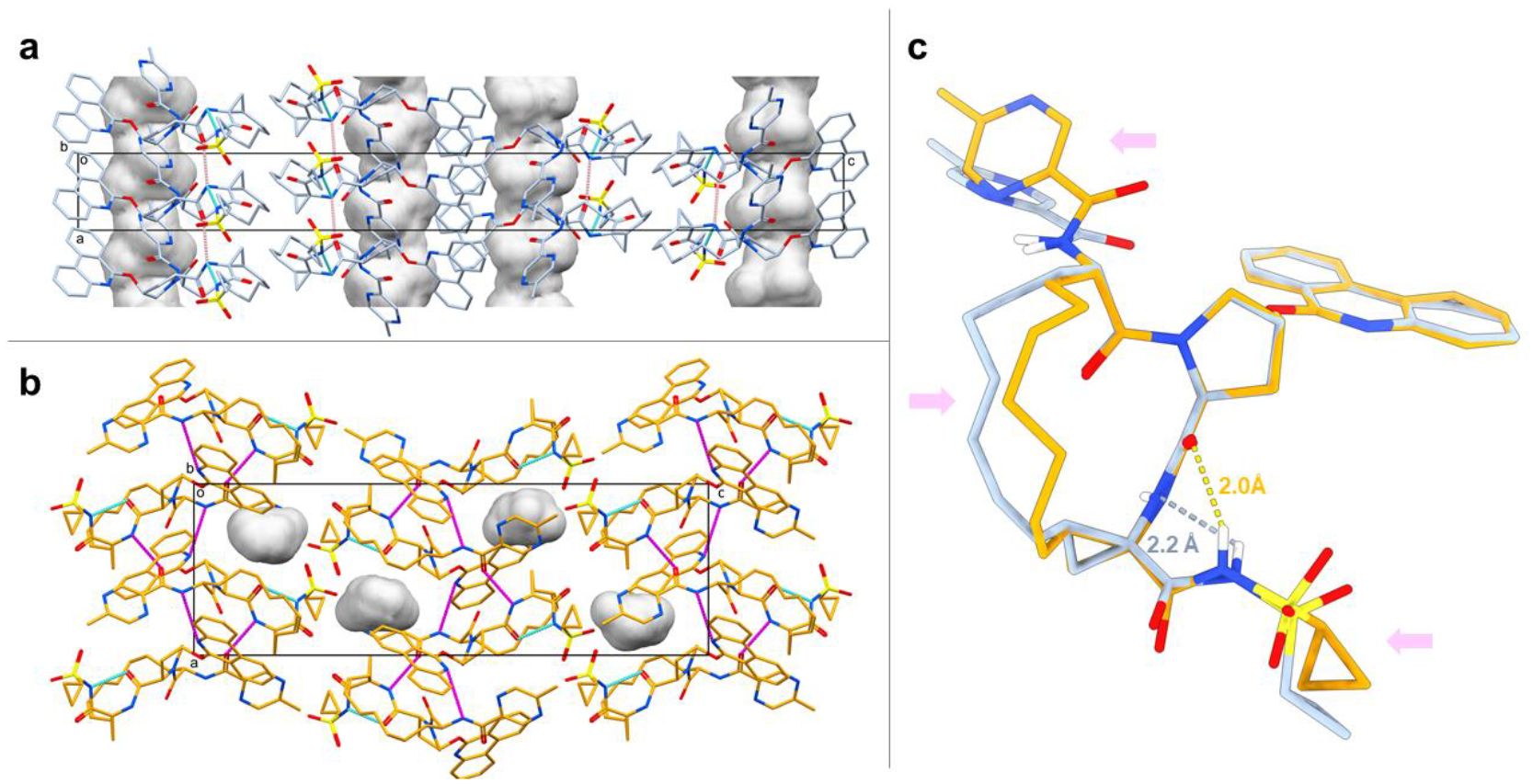
**a**: Unit cell packing of paritaprevir form α viewed along the crystallographic b axis with the unit cell box shown in black. Atom colors: C, light blue; N, blue; O, red; S, yellow. The inter- and intramolecular hydrogen bonds are shown in magenta and cyan dashed lines, respectively. The one-dimensional solvent channels are shown in gray contoured surface extending along the crystallographic a axis. All hydrogens are omitted for clarity. **b**: Unit cell packing of paritaprevir form β viewed along the crystallographic b axis with the unit cell box shown in black. Atom colors: C, orange; N, blue; O, red; S, yellow. The inter- and intramolecular hydrogen bonds are shown in magenta and blue dashed lines, respectively. The voids are shown in gray contoured surface. All hydrogens are omitted for clarity. **c**: Overlay of all heavy atoms on the aromatic rings, 5-membered ring and macrocyclic core amides between paritaprevir form α (light blue) and form β (orange), showing conformational changes in the macrocyclic core, methyl pyrazinamide moiety and cyclopropyl sulfonamide moiety, as indicated by the pink arrows. The intramolecular hydrogen bonds are shown in dashed lines. Non-polar hydrogens are omitted for clarity.

Paritaprevir form α adopts an open conformation with one intramolecular hydrogen bond between the amide nitrogen on the macrocyclic core and the cyclopropyl sulfonamide moiety (2.2 Å, Figure 2a and c). The packing analysis reveals that intermolecular hydrogen bonds between the amide carbonyls and amide nitrogens on the macrocyclic cores generate chain motifs along the crystallographic a axis. Further, the phenanthridine rings form intermolecular π-π interactions (Figure 2a and S2). The packing of form α gives rise to the formation of solvent-accessible channels which form along the crystallographic a axis (Figure 2a). Paritaprevir form β adopts another open conformation with an intramolecular hydrogen bond between the amide carbonyl on the macrocyclic core and amide nitrogen on the cyclopropyl sulfonamide moiety (2.0 Å, Figure 2b and c). As compared to form α, the packing of form β differs; intermolecular hydrogen bonds between the amide carbonyl on the methyl pyrazinamide moiety and amide nitrogen on the macrocyclic core, as well as between amide nitrogen on the methyl pyrazinamide moiety and aromatic nitrogen on the phenanthridine ring, generates chain motifs along the crystallographic a axis (Figure 2b and S4).

The observation of solvent channels in pharmaceutical crystals of molecules with chameleonic behavior has been previously described [16, 29] and can lead to reduced aqueous solubility [29] and risks in processing, manufacturing and storage of the drugs. [30] Using the default void analysis in Mercury with a probe radius of 1.2 Å, 7.6% of the unit cell for form α is concluded to consist of solvent-accessible channels extending along the crystallographic a axis (Figures 2a and S2). Paritaprevir form β does not have the large channels observed for form α. Instead, the crystal packing is significantly tighter with only 2.2% of the unit cell corresponding to discrete void space (Figure 2b and S4). The space group and unit cell dimensions of form α match those of a paritaprevir hydrate crystal form studied by powder X-ray diffraction (PXRD), [16, 17] with the unit cell axis of a and c being flipped. In the PXRD study, water molecules are located in the one-dimensional channels and interact with each other, with the sulfonamide oxygen, and with the phenanthridine nitrogen. Although the PXRD structure is not publicly accessible, form α shares a similar crystal packing with the paritaprevir hydrate form based on the same space group, unit cell dimensions, and the one-dimensional channels extending along the crystallographic axis. In contrast to the PXRD study, paritaprevir form α is solved as an anhydrate, suggesting that it can retain the crystallinity during hydration/dehydration [17] and hence risk trapping water molecules upon storage. Taken together with the observed strong intermolecular interactions, form α is therefore assumed to have unfavorable properties for storage, solubility and dissolution rate in water, and thus is not a good candidate for formulation and process development. [16]

Although both form α and form β adopt open conformations, the structures are different with an RMSD value of 0.83 Å comparing all heteroatoms (Figure 2c). The most significant conformational changes include the alkyl chain motifs of the macrocyclic core, with a torsion angle difference over 90°, the different rotamers of the methyl pyrazinamide substituents, and the cyclopropyl sulfonamide moieties with slightly different orientations and significant change in torsion, resulting in different intramolecular hydrogen bond locations (Figure 2c). The calculated solvent accessible three-dimensional polar surface area (SA 3D PSA) are 175.13 Å^2^ and 188.28 Å^2^ for form α and form β, respectively. The latter compares well to the 3D-PSA value of a simulated target-bound paritaprevir conformer (186.7 Å^2^). [16] In order to understand the biological importance of both conformers, molecular docking was applied to the protein target.

### Molecular docking.

**Figure 3.**
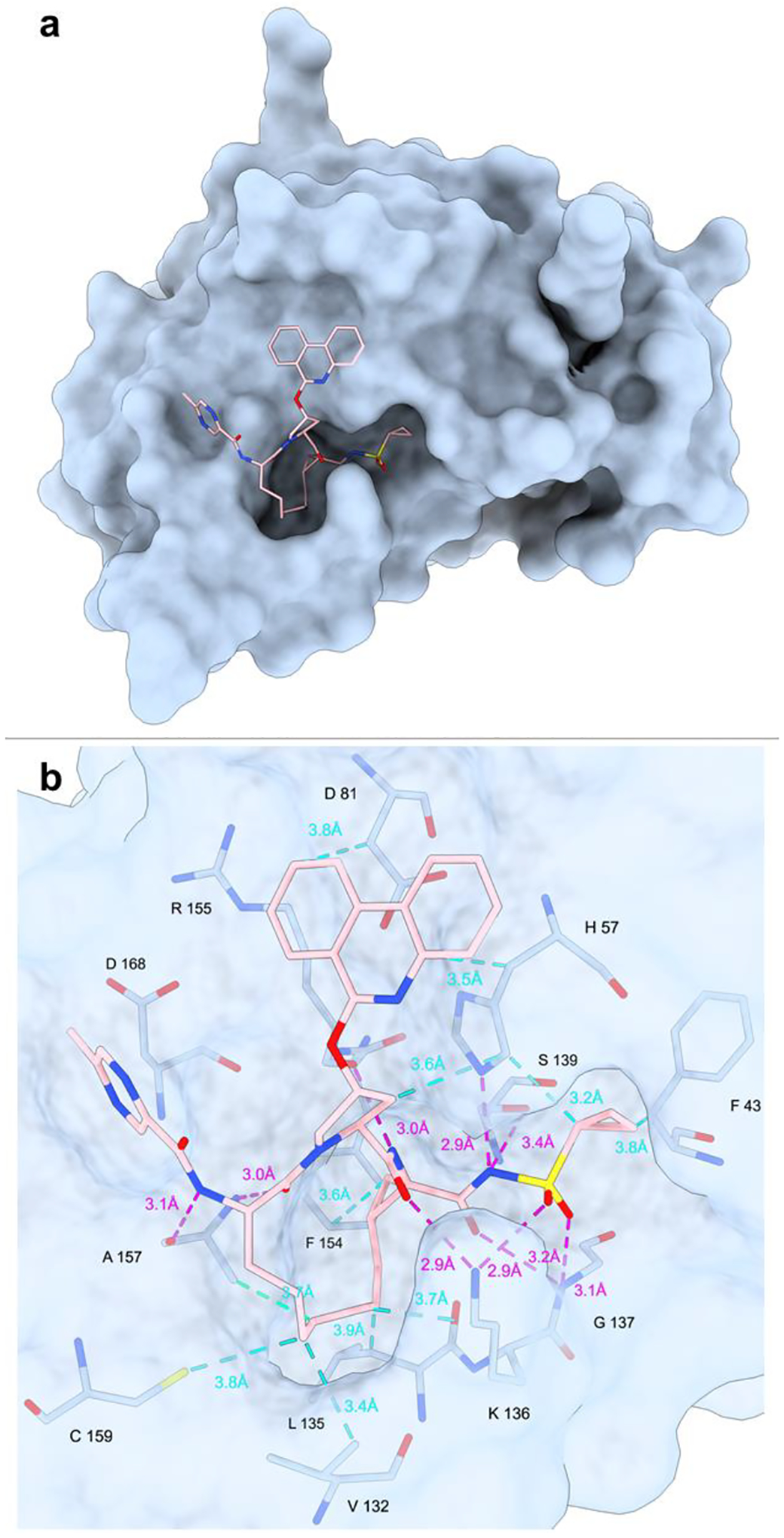
**a**: Docking of paritaprevir conformer β into the simeprevir-bound crystal structure of HCV NS3/4A protease (PDB ID: 3KEE). Atom colors: C, pink; N, blue; O, red; S, yellow. All hydrogen atoms are omitted for clarity **b:** Potential **i**nteractions between paritaprevir and the target observed from the molecular docking. The intermolecular hydrogen bonds and hydrophobic interactions are shown as dashed lines in magenta and cyan, respectively.

Molecular docking experiments of paritaprevir conformers α and β into HCV NS3/4A protease were performed using Glide [31] and the crystal structure of simeprevir-bound genotype 1b HCV NS3/4A (PDB ID: 3KEE [32]) as the receptor, in which the coordinates of simeprevir, water molecules, and other ligands were removed and hydrogens were added to all protein residues. This structure was selected because simeprevir (Figure S5) and paritaprevir are structurally similar and both target the HCV NS3/4A protease. The docking procedure was validated by using the simeprevir structure from the PDB entry 3KEE [32] as ligand, which was docked to the active site with a docking score of −8.2 kcal/mol and an RMSD value of 0.28 Å, comparing the experimental and docked simeprevir. Conformer α docked outside of the active site of the protease (result not shown), with a docking score of −4.2 kcal/mol, and conformer β docked into the active site pocket with a docking score of −11.3 kcal/mol (Figure 3a and b). [33–35] Hence, conformer β is more thermodynamically favored for target binding. The simeprevir-bound crystal structure revealed the antiviral to disrupt the catalytic triad of the HCV NS3/4A protease (H57, D81 and S139) by forming hydrogen bonds to side chains S139 and H57 (Figure S5). Additional hydrogen bonds are formed between simeprevir and the backbones of G137, R155 and A157, and between simeprevir and the side chain of K136, where the latter serves as a clamp to close the active site pocket (Figure S5). The structure-activity relationship study of paritaprevir and the HCV protease revealed the macrocyclic core, the cyclopropyl sulfonamide moiety and the phenanthridine ring to be of high importance for the inhibitory activity. [14] The macrocyclic core interacts extensively with the pocket, similar to simeprevir, via several hydrophobic interactions as well as via hydrogen bonds to the side chain of K136, and to the backbones of R155 and A157 (Figure 3b and Table S2). The cyclopropyl sulfonamide has long been incorporated into other drugs for HCV NS3/4A protease target. [14] In our model, paritaprevir interacts with the catalytic triad through hydrogen bonds between the cyclopropyl sulfonamide and the side chains of H57 and S139 (Figure 3b and Table S2), where the former hydrogen bond has been reported in previous docking simulations [16]. In addition, this substituent makes a hydrogen bond to the K136 side chain, which is not present in the simeprevir structure, as well as to the G137 backbone, which has been reported in previous docking simulations [16]. K136 forms a clamp to control the opening of the active site pocket, and G137 is the site of the oxyanion hole. [36] The phenanthridine ring of paritaprevir shows hydrophobic interaction with the side chains of H57 and D81, where the latter is not present in the simeprevir-bound structure. The 5-membered ring linking the phenanthridine to the core shows additional hydrophobic interaction with the side chain of H57. Lastly, the methyl pyrazinamide moiety interacts with the target via a hydrogen bond to the backbone of A157 (Figure 3b and Table S2). The few interactions of this substituent corroborate its moderate effect in improving potency, however it was shown to be important for metabolic stability and pharmacokinetics. [14] It can be noted that the potency of paritaprevir for the HCV genotype 1b target (EC_50_ = 0.21 nM) [14] is higher than for simeprevir (EC_50_ = 25 nM), [37] which might partly be explained by some of additional target interactions for paritaprevir suggested here.

## Conclusions

The development of high-throughput MicroED with automation has recently been successfully applied in drug discovery for the analysis of small molecule therapeutics, [28, 38, 39] and for the structural determination of large and flexible macrocycles. [19] Herein, high-throughput MicroED is successfully used for polymorph screening from the same experiment, without crystallization assays or different sample preparations. Paritaprevir is known to have chameleonic properties and adopts open conformations in aqueous environment and closed conformations in lipophilic environment. Two of the conformations could be determined from the same CryoEM grid where one is suggested to be the target bound state. We show that high-throughput MicroED will be suitable for future polymorph screening in future drug discovery, and that MicroED structures of complex modalities in the beyond rule of 5 space can be used for precision docking to difficult-to-drug targets.

## Supplementary Material

Supinfo

## References

[1] Byrn SR, , Solid-State Pharmaceutical Chemistry. Chemistry of Materials, 1994. 6(8): p. 1148–1158.

[2] Lu J and Rohani S, Polymorphism and crystallization of active pharmaceutical ingredients (APIs). Curr Med Chem, 2009. 16(7): p. 884–905. 19275600 10.2174/092986709787549299

[3] Bruhn JF, , Small Molecule Microcrystal Electron Diffraction for the Pharmaceutical Industry–Lessons Learned From Examining Over Fifty Samples. Frontiers in Molecular Biosciences, 2021. 8. 10.3389/fmolb.2021.648603 PMC8313502 34327213

[4] Morissette SL, , High-throughput crystallization: polymorphs, salts, co-crystals and solvates of pharmaceutical solids. Advanced Drug Delivery Reviews, 2004. 56(3): p. 275–300. 14962582 10.1016/j.addr.2003.10.020

[5] Cruz-Cabeza AJ, Reutzel-Edens SM, and Bernstein J, Facts and fictions about polymorphism. Chemical Society Reviews, 2015. 44(23): p. 8619–8635. 26400501 10.1039/c5cs00227c

[6] Bauer J, , Ritonavir: an extraordinary example of conformational polymorphism. Pharm Res, 2001. 18(6): p. 859–66. 11474792 10.1023/a:1011052932607

[7] Driggers EM, , The exploration of macrocycles for drug discovery — an underexploited structural class. Nature Reviews Drug Discovery, 2008. 7(7): p. 608–624. 18591981 10.1038/nrd2590

[8] Lipinski CA, , Experimental and computational approaches to estimate solubility and permeability in drug discovery and development settings. Advanced Drug Delivery Reviews, 1997. 23(1): p. 3–25. 10.1016/s0169-409x(00)00129-0 11259830

[9] Doak BC, , Oral druggable space beyond the rule of 5: insights from drugs and clinical candidates. Chem Biol, 2014. 21(9): p. 1115–42. 25237858 10.1016/j.chembiol.2014.08.013

[10] Blanco M-J and Gardinier KM, New Chemical Modalities and Strategic Thinking in Early Drug Discovery. ACS Medicinal Chemistry Letters, 2020. 11(3): p. 228–231. 32184948 10.1021/acsmedchemlett.9b00582 PMC7073867

[11] Cruz-Cabeza AJ and Bernstein J, Conformational Polymorphism. Chemical Reviews, 2014. 114(4): p. 2170–2191. 24350653 10.1021/cr400249d

[12] DeGoey DA, , Beyond the Rule of 5: Lessons Learned from AbbVie’s Drugs and Compound Collection. Journal of Medicinal Chemistry, 2018. 61(7): p. 2636–2651. 28926247 10.1021/acs.jmedchem.7b00717

[13] Pilot-Matias T, , In vitro and in vivo antiviral activity and resistance profile of the hepatitis C virus NS3/4A protease inhibitor ABT-450. Antimicrob Agents Chemother, 2015. 59(2): p. 988–97. 25451053 10.1128/AAC.04227-14 PMC4335891

[14] McDaniel KF, , The Discovery and Development of HCV NS3 Protease Inhibitor Paritaprevir, in HCV: The Journey from Discovery to a Cure: Volume I, Sofia MJ, Editor. 2019, Springer International Publishing: Cham. p. 389–413.

[15] Mensing S, , Population pharmacokinetics of paritaprevir, ombitasvir, dasabuvir, ritonavir and ribavirin in hepatitis C virus genotype 1 infection: analysis of six phase III trials. Br J Clin Pharmacol, 2017. 83(3): p. 527–539. 27662429 10.1111/bcp.13138 PMC5306483

[16] Sheikh AY, , Implications of the Conformationally Flexible, Macrocyclic Structure of the First-Generation, Direct-Acting Anti-Viral Paritaprevir on Its Solid Form Complexity and Chameleonic Behavior. Journal of the American Chemical Society, 2021. 143(42): p. 17479–17491. 34637297 10.1021/jacs.1c06837

[17] Hong RS, , Distinct Hybrid Hydrates of Paritaprevir: Combined Experimental and Computational Assessment of their Hydration–Dehydration Behavior and Implications for Regulatory Controls. Crystal Growth & Design, 2022. 22(1): p. 726–737.

[18] Caspi DD, , Process development of ABT-450–A first generation NS3/4A protease inhibitor for HCV. Tetrahedron, 2019. 75(32): p. 4271–4286.

[19] Danelius E, , MicroED as a powerful tool for structure determination of macrocyclic drug compounds directly from their powder formulations. bioRxiv, 2023, doi: 10.1101/2023.07.31.551405. PMC10728894 37944119

[20] Shi D, , Three-dimensional electron crystallography of protein microcrystals. eLife, 2013. **2**: p. e01345. 24252878 10.7554/eLife.01345 PMC3831942

[21] Nannenga BL, , High-resolution structure determination by continuous-rotation data collection in MicroED. Nature Methods, 2014. 11(9): p. 927–930. 25086503 10.1038/nmeth.3043 PMC4149488

[22] Nannenga BL and Gonen T, The cryo-EM method microcrystal electron diffraction (MicroED). Nature Methods, 2019. 16(5): p. 369–379. 31040436 10.1038/s41592-019-0395-x PMC6568260

[23] Broadhurst ET, , Polymorph evolution during crystal growth studied by 3D electron diffraction. IUCrJ, 2020. 7(1): p. 5–9. 10.1107/S2052252519016105 PMC6949601 31949899

[24] Levine AM, , Efficient Free Triplet Generation Follows Singlet Fission in Diketo-pyrrolopyrrole Polymorphs with Goldilocks Coupling. J Phys Chem C Nanomater Interfaces, 2021. 125(22): p. 12207–12213. 34868444 10.1021/acs.jpcc.1c02737 PMC8641251

[25] Lightowler M, , Indomethacin Polymorph δ Revealed To Be Two Plastically Bendable Crystal Forms by 3D Electron Diffraction: Correcting a 47-Year-Old Misunderstanding**. Angewandte Chemie International Edition, 2022. 61(7): p. e202114985. 34902212 10.1002/anie.202114985 PMC9306882

[26] Cho HJ, , Microcrystal Electron Diffraction Elucidates Water-Specific Polymorphism-Induced Emission Enhancement of Bis-arylacylhydrazone. ACS Applied Materials & Interfaces, 2021. 13(6): p. 7546–7555. 33544590 10.1021/acsami.0c21248

[27] Li S, , Direct structure determination of vemurafenib polymorphism from compact spherulites using 3D electron diffraction. Communications Chemistry, 2023. 6(1): p. 18. 36697943 10.1038/s42004-022-00804-2 PMC9871043

[28] Unge J, , A Autonomous MicroED data collection enables compositional analysis. 2023, doi: 10.26434/chemrxiv-2023-8qvwg.

[29] Pereira BG, , Pseudopolymorphs and Intrinsic Dissolution of Nevirapine. Crystal Growth & Design, 2007. 7(10): p. 2016–2023.

[30] Takahashi M and Uekusa H, Dehydration and Rehydration Mechanisms of Pharmaceutical Crystals: Classification Of Hydrates by Activation Energy. Journal of Pharmaceutical Sciences, 2022. 111(3): p. 618–627. 34728174 10.1016/j.xphs.2021.10.033

[31] Friesner RA, , Glide: A New Approach for Rapid, Accurate Docking and Scoring. 1. Method and Assessment of Docking Accuracy. Journal of Medicinal Chemistry, 2004. 47(7): p. 1739–1749. 15027865 10.1021/jm0306430

[32] Cummings MD, , Induced-Fit Binding of the Macrocyclic Noncovalent Inhibitor TMC435 to its HCV NS3/NS4A Protease Target. Angewandte Chemie International Edition, 2010. 49(9): p. 1652–1655. 20166108 10.1002/anie.200906696

[33] Friesner RA, , Extra Precision Glide: Docking and Scoring Incorporating a Model of Hydrophobic Enclosure for Protein−Ligand Complexes. Journal of Medicinal Chemistry, 2006. 49(21): p. 6177–6196. 17034125 10.1021/jm051256o

[34] Yewale SB, , Novel 3-substituted-1-aryl-5-phenyl-6-anilinopyrazolo[3,4-d]pyrimidin-4-ones: docking, synthesis and pharmacological evaluation as a potential anti-inflammatory agents. Bioorg Med Chem Lett, 2012. 22(21): p. 6616–20. 23036953 10.1016/j.bmcl.2012.08.119

[35] Arun KG, , Drug repurposing against SARS-CoV-2 using E-pharmacophore based virtual screening, molecular docking and molecular dynamics with main protease as the target. J Biomol Struct Dyn, 2021. 39(13): p. 4647–4658. 32571168 10.1080/07391102.2020.1779819 PMC7335810

[36] Hedstrom L, Serine Protease Mechanism and Specificity. Chemical Reviews, 2002. 102(12): p. 4501–4524. 12475199 10.1021/cr000033x

[37] Lin TI, , In vitro activity and preclinical profile of TMC435350, a potent hepatitis C virus protease inhibitor. Antimicrob Agents Chemother, 2009. 53(4): p. 1377–85. 19171797 10.1128/AAC.01058-08 PMC2663092

[38] Lin J, , Distinct Conformations of Mirabegron Determined by MicroED. bioRxiv, 2023, doi: 10.1101/2023.06.28.546957. PMC10700164 37847906

[39] Gogoi DST, , Structure elucidation of olanzapine molecular salts by combining mechanochemistry and MicroED. Cryst. Growth Des 2023, 23:8, 5821–5826.

