# Supplementary material for: Polymorphic Structure Determination of the Macrocyclic Drug Paritaprevir by MicroED
Source: Adv Biol (Weinh). Author manuscript; Available in PMC 2024 May 14. (PMC11090733; doi:10.1002/adbi.202300570)
Supplement: Supinfo [file NIHMS1968377-supplement-Supinfo.docx]

**Polymorphic Structure Determination of** **the Macrocyclic Drug Paritaprevir by MicroED**

Guanhong Bu^1,+^, Emma Danelius^1,2,+^, Lianne H.E. Wieske^3^, and Tamir Gonen*^1,2,4^

1. Department of Biological Chemistry, University of California Los Angeles, 615 Charles E. Young Drive South, Los Angeles, CA 90095, USA.
2. Howard Hughes Medical Institute, University of California Los Angeles, Los Angeles, CA 90095, USA.
3. Department of Chemistry – BMC, Uppsala University, Husargatan 3, 75237 Uppsala, Sweden.
4. Department of Physiology, University of California Los Angeles, 615 Charles E. Young Drive South, Los Angeles, CA 90095, USA.

+ Denotes equal contribution

* Correspondence E-mail: [tgonen@g.ucla.edu](mailto:tgonen@g.ucla.edu); ORCID: https://orcid.org/0000-0002-9254-4069

**Table of contents**

Experimental 2

Sample preparation 2

MicroED data collection 2

MicroED data processing and structure determination 2

Molecular docking 2

Supplementary figures 3

Supplementary tables 8

References 10

**Experimental**

**Sample preparation.** Approximately 0.5 mg paritaprevir (Invivochem) was dissolved into minimal amounts of methanol in a clean 4 mL scintillator vial. To prepare microcrystals, the lid was kept open in a fume hood and the methanol was slowly evaporated at room temperature for approximately 20 h. To transfer the microcrystals onto the TEM grid, the resulting dry deposits were scraped from the glass wall, followed by adding the pre-clipped 400-mesh TEM grid (Ted Pella) into the scintillator vial and gently shaking the vial. Prior to mixing with crystals, the TEM grid was coated with continuous carbon film and treated by glow-discharging for 30 seconds on each side at 15 mA on the negative mode using PELCO easiGlow (Ted Pella).

**MicroED data collection.** The TEM grid was loaded into a Thermo-Fisher Talos Arctica electron microscope operating at 80 K and 200 kV (0.0251 Å wavelength). MicroED datasets were automatically collected using SerialEM following the published protocol in our recent work [1]. The whole grid atlas was acquired as a low-magnification montage at a magnification of 155x. Grid squares containing microcrystals were selected by the SerialEM “Navigator”, and acquired for medium-magnification montages at a magnification of 2,600x. During the medium-magnification montage, the “Fine eucentricity” function in SerialEM was selected to assign the eucentric heights for each grid square in the corresponding maps. Microcrystals were picked from each medium-magnification montage within the “Navigator” window for data collection. MicroED data collection was performed in the SerialEM “Record” mode where the microscope was set for the parallel electron diffraction settings (C2 lens intensity of 45.2% inserted with an aperture size of 20, resulting in the beam size of approximately 1.5 µm in diameter). Continuous-rotation MicroED data were recorded in MRC format on a Thermo-Fisher Falcon III detector in linear mode at an electron dose rate of 0.01 e^-^ Å^-2^ s^-1^ and 1 second exposure per frame as the sample stage was tilting from -30˚ to +30˚ at 1˚ per second.

**MicroED data processing and structure determination.** The datasets were initially processed using an in-house developed python script for automatic image conversion, indexing, integration and scaling [1, 2]. The information on completeness, resolution and cell dimensions generated by our automatic script provided a guideline for which data sets could be manually processed and merged in XDS [3] in order to improve the processing and merging statistics. Reflection files were prepared using XPREP (Bruker), and the *ab initio* structures were determined by SHELXD [4], followed by refinement in SHELXL [5] using electron scattering factors. Unless specified, hydrogen atoms were located at the geometrically idealized positions and refined using riding model. MicroED structures were visualized in ChimeraX [6]. Crystal packing and RMSD were analyzed in Mercury [7]. Solvent accessible 3D polar surface area (SA 3D PSA) for each conformation was calculated in PyMOL using a solvent probe radius of 1.4 Å as previously described [8, 9].

**Molecular docking.** Molecular docking was performed in Maestro (Schrodinger) using the published crystal structure of simeprevir-bound Hepatitis C virus (HCV) NS3/4A protease (PDB ID: 3KEE [10]) as the receptor. At first, the default settings of Protein Preparation Wizard were employed for preparing the receptor molecule for docking. Simeprevir, water and other ligand molecules were removed from the receptor. Hydrogens were added to the receptor and optimized using the PROPKA algorithm. The receptor was then energy-minimized using the OPLS4 force field. The next procedure was to generate the receptor grid for docking using a box size of 36 Å in length centered at the H57 residue of the NS3 chain. The van der Waals radii of nonpolar receptor atoms were scaled at a factor of 1.0 using a partial charge cutoff at 25%. Lastly, docking experiments were performed using Glide (Schrodinger) [11] in the extra precision (XP) mode. Paritaprevir conformer α and β were set as flexible. Nitrogen inversions and the macrocycle ring conformations were sampled. The van der Waals radii of nonpolar ligand atoms were scaled at a factor of 0.8 using a partial charge cutoff at 30%. Conformer α docked outside of the active site pocket, with a score of -4.2 kcal/mol. Conformer β docked into the active site pocket, with a score of -11.3 kcal/mol. The docking procedure was validated by a control study; the target-bound structure of simeprevir (PDB ID: 3KEE [10]) was re-docked to the pre-processed and prepared protein using the same grid box and settings as for paritaprevir conformer α and β. The docking score was used as a standard value. Simeprevir was docked into the target as observed in the X-ray structure [10] with a docking score of -8.2 kcal/mol and an RMSD value of 0.28 Å, comparing the experimental and calculated simeprevir. The docked model and protein-drug interactions were visualized in ChimeraX [6].

**
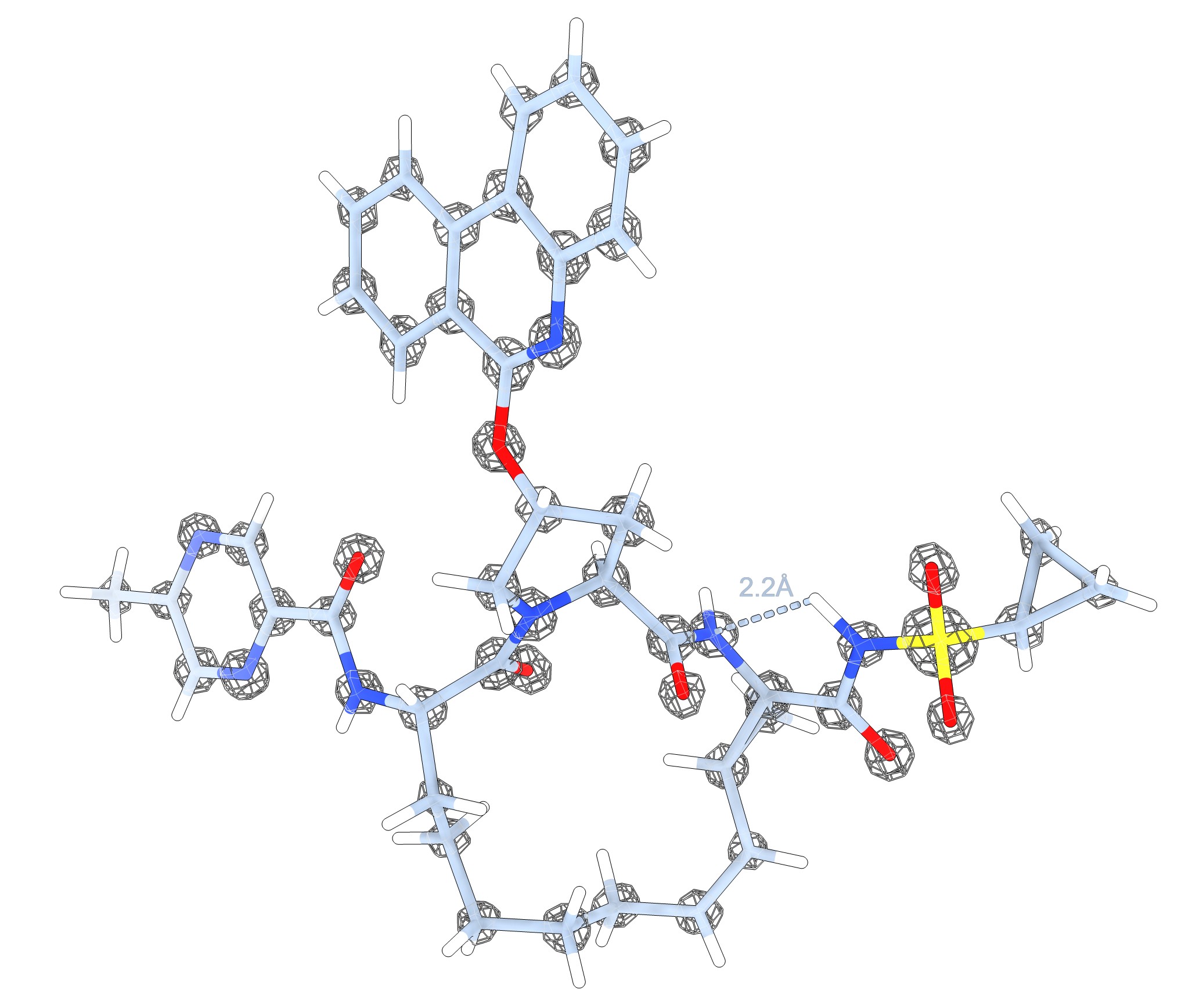
**

**Figure S1:** MicroED structure of paritaprevir form α with the density contoured at 1.5σ level. Atom color: C, gray; N, blue; O, red; S, yellow; H, white. Densities are shown as meshed contoured surface in light steel blue.

**
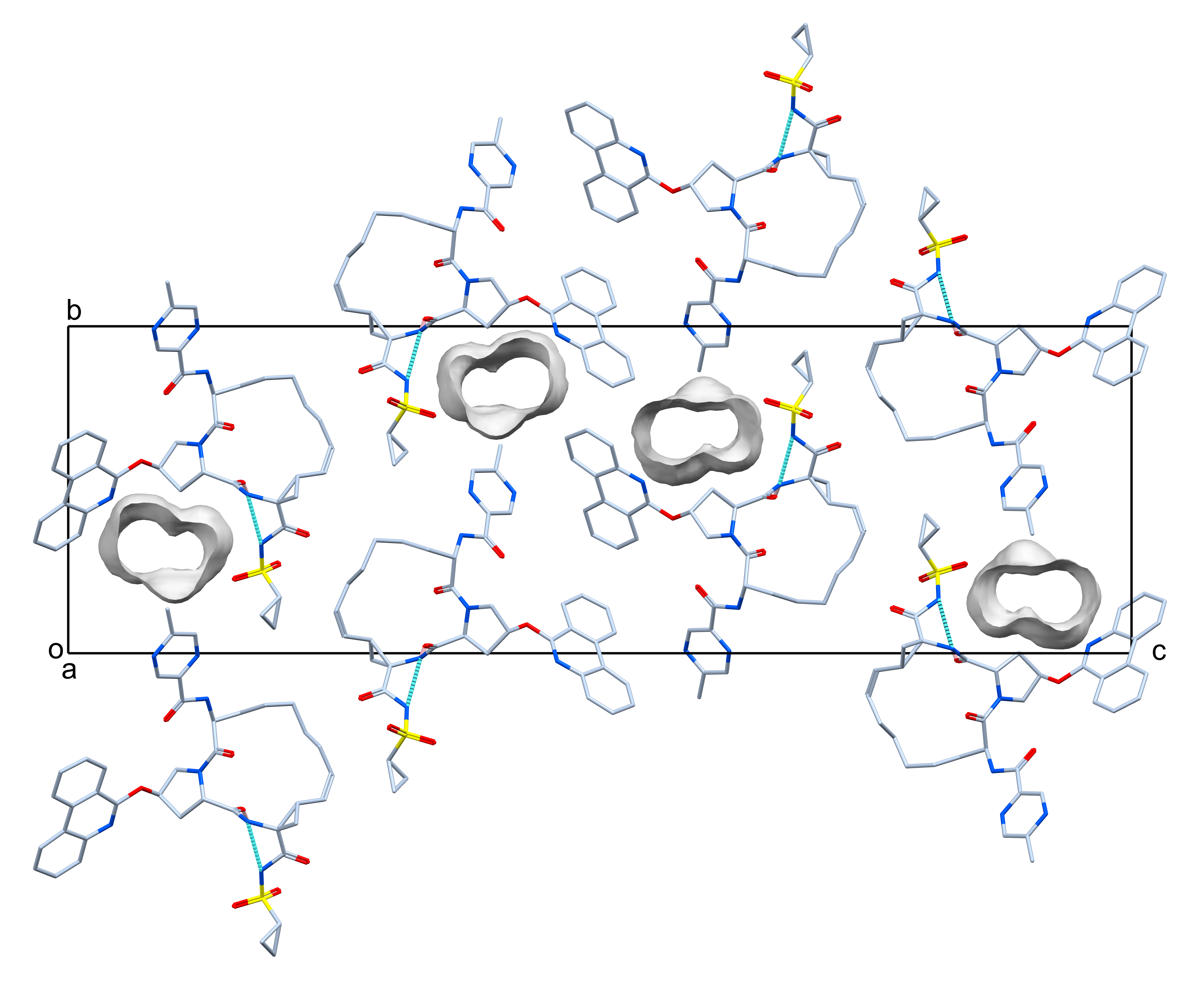
**

**Figure S2:** Unit cell packing of paritaprevir form α viewed along the crystallographic a axis with the unit cell box shown in black. Atom colors: C, light steel blue; N, blue; O, red; S, yellow. The inter- and intramolecular hydrogen bonds are shown in magenta and cyan dashed lines, respectively. The one-dimensional channels are shown in light gray contoured surface extending along the crystallographic a axis. All hydrogens are omitted for clarity.

**
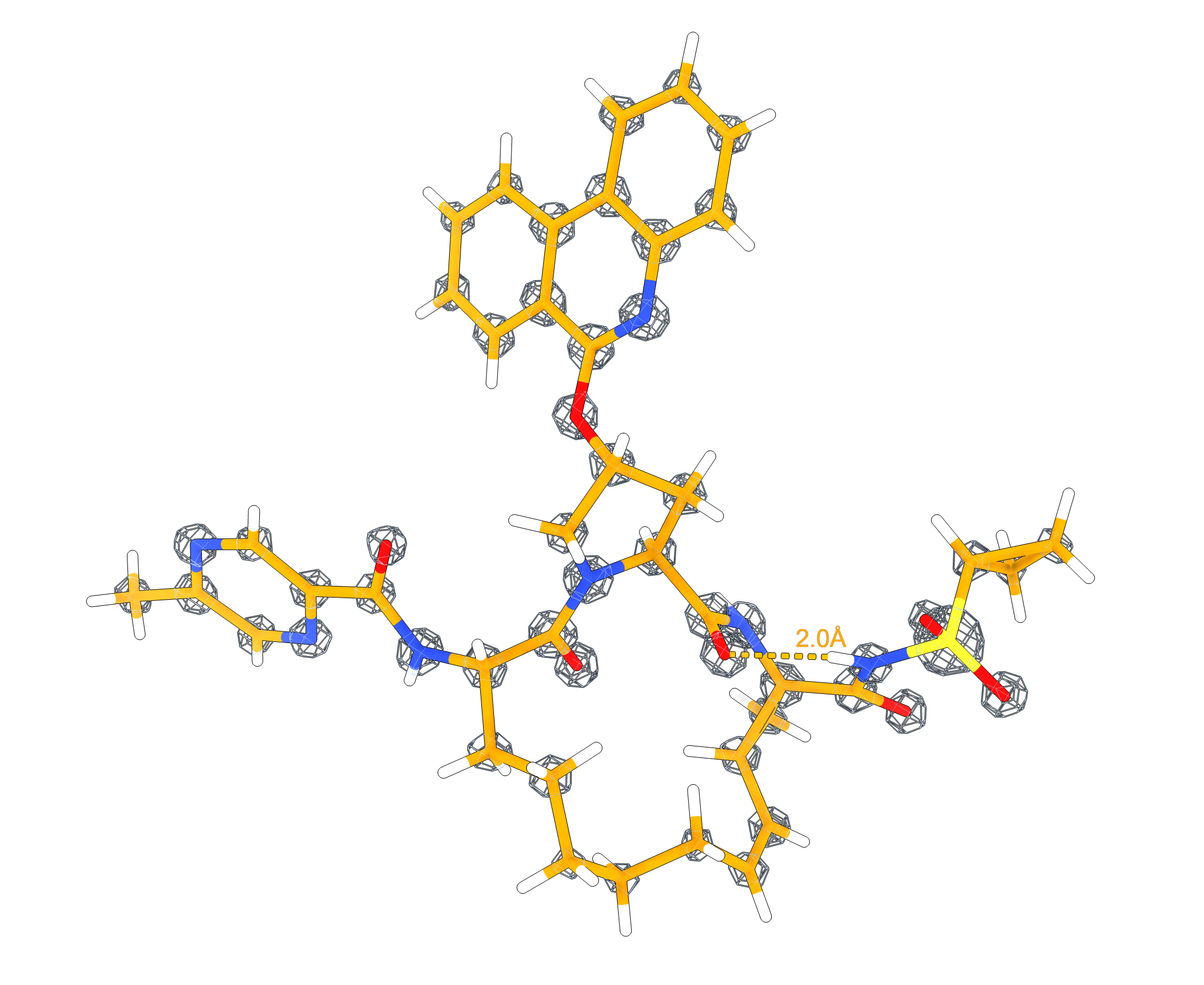
**

**Figure S3:** MicroED structure of paritaprevir form β with the density contoured at 1.5σ level. Atom color: C, orange; N, blue; O, red; S, yellow; H, white. Densities are shown as meshed contoured surface in light steel blue.

**
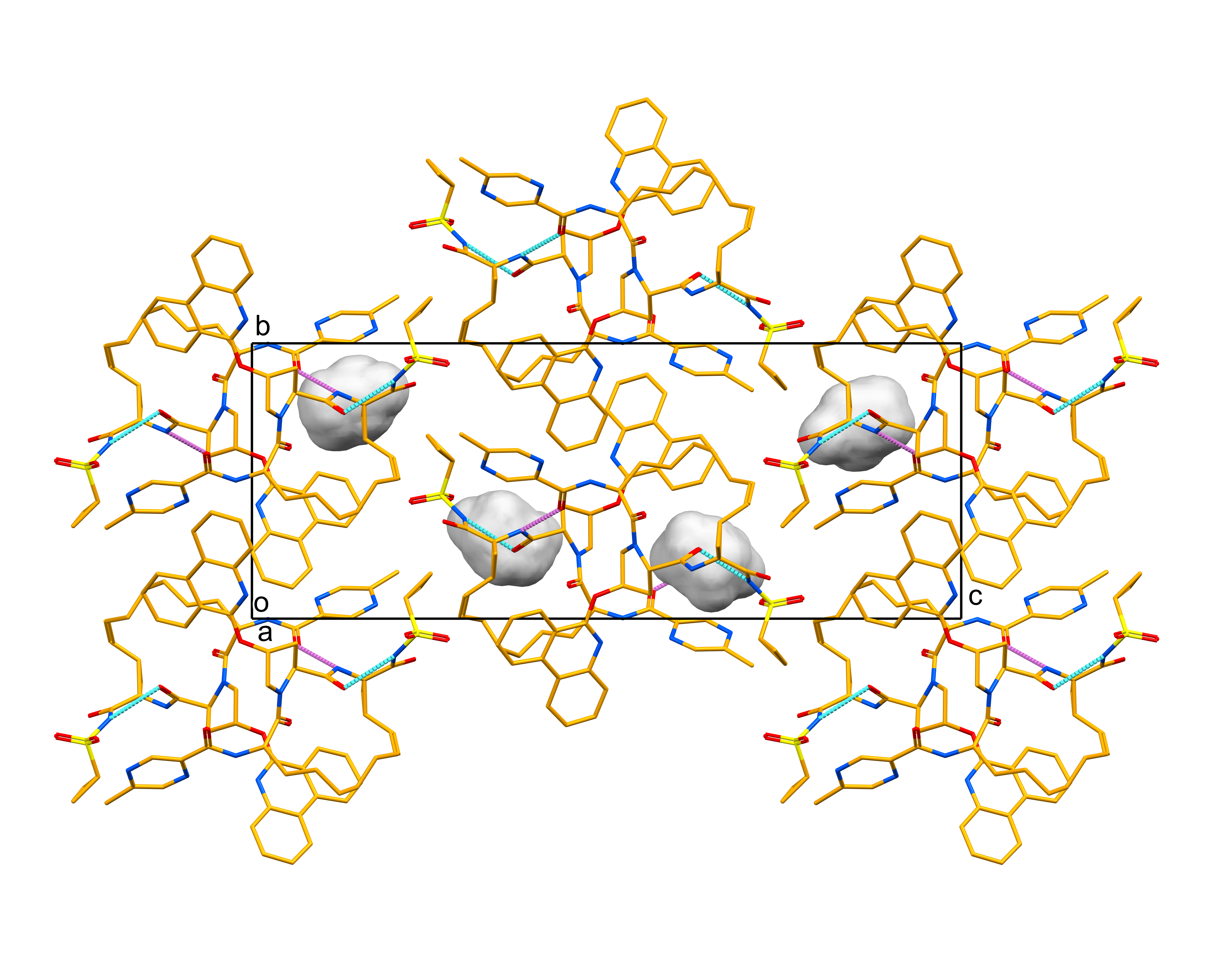
**

**Figure S4:** Unit cell packing of paritaprevir form β viewed along the crystallographic a axis with the unit cell box shown in black. Atom colors: C, orange; N, blue; O, red; S, yellow. The inter- and intramolecular hydrogen bonds are shown in magenta and cyan dashed lines, respectively. The voids are shown in light gray contoured surface. All hydrogens are omitted for clarity.

**
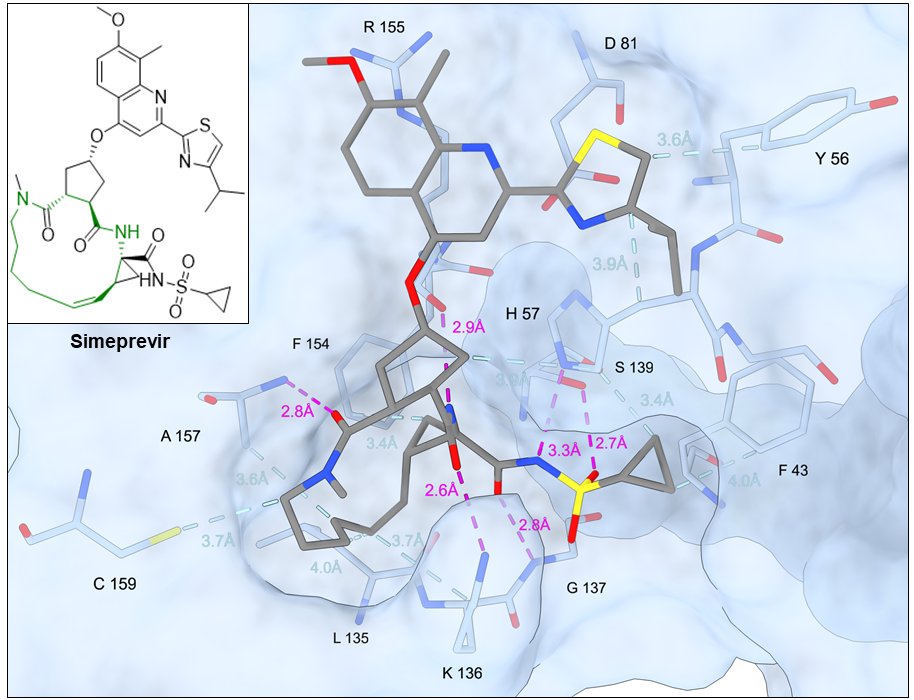
**

**Figure S5:** Interactions between simeprevir and HCV NS3/4A protease observed from the X-ray structure (PDB ID: 3KEE) [10]. Atom color in simeprevir: C, gray; N, blue; O, red; S, yellow. Hydrophobic interactions and hydrogen bonds are shown as light blue and magenta dashed lines, respectively. All hydrogen atoms are omitted for clarity.

**Table S1**. Processing and structure refinement statistics of paritaprevir form α and β

|  | **Form α** | **Form β** |
| --- | --- | --- |
| Stoichiometric formula | C_40_H_43_N_7_O_7_S | C_40_H_43_N_7_O_7_S |
| Radiation wavelength (Å) | 0.0251 | 0.0251 |
| Temperature (K) | 80 | 80 |
| Number of crystals | 2 | 2 |
| Crystal shape | needle | rod |
| Resolution (Å) | 50.0 - 0.85 | 50.0 - 0.95 |
| Crystal system | orthorhombic | orthorhombic |
| Space group | P2_1_2_1_2_1_ | P2_1_2_1_2_1_ |
| Unit cell length a, b, c (Å) | 5.09, 15.61, 50.78 | 10.56, 12.32, 31.73 |
| Unit cell angle α, β, ɣ (°) | 90, 90, 90 | 90, 90, 90 |
| Z | 4 | 4 |
| Total reflections | 15,086 | 16,900 |
| Unique reflections | 3,607 | 2,851 |
| R_obs_ (%) | 20.2 | 22.8 |
| R_meas_ (%) | 23.2 | 24.9 |
| I/σΙ | 5.20 | 5.41 |
| CC_1/2_ (%) | 98.9 | 98.9 |
| Completeness (%) | 89.0 | 98.4 |
| R_1_ | 0.1472 | 0.1347 |
| wR_2_ | 0.4080 | 0.3734 |
| GooF | 1.156 | 1.114 |
| **CCDC deposition number** | **2286074** | **2294331** |

**Table S2**. Statistics of interactions between paritaprevir and HCV NS3/4A protease observed from molecular docking.

| **Interactions** | **Receptor atom (residue)** | **Ligand atom (moiety)** | **Distance in Å** |
| --- | --- | --- | --- |
| **Hydrogen bond** | NE2 (H57) | N3 (cyclopropyl sulfonamide) | 2.9 |
|  | NZ (K136) | O2 (macrocyclic core) | 2.9 |
|  | NZ (K136) | O5 (cyclopropyl sulfonamide) | 2.9 |
|  | N (G137) | O4 (cyclopropyl sulfonamide) | 3.1 |
|  | N (G137) | O3 (cyclopropyl sulfonamide) | 3.2 |
|  | OG (S139) | N3 (cyclopropyl sulfonamide) | 3.4 |
|  | O (R155) | N2 (macrocyclic core) | 3.0 |
|  | N (A157) | O1 (macrocyclic core) | 3.0 |
|  | O (A157) | N4 (methyl pyrazinamide) | 3.1 |
| **Hydrophobic interaction** | CB (F43) | C26 (cyclopropyl sulfonamide) | 3.8 |
|  | CD2 (H57) | C25 (cyclopropyl sulfonamide) | 3.2 |
|  | CB (H57) | C40 (phenanthridine) | 3.5 |
|  | CD2 (H57) | C3 (5-membered core) | 3.6 |
|  | CB (D81) | C32 (phenanthridine) | 3.8 |
|  | CG1 (V132) | C13 (macrocyclic core) | 3.4 |
|  | CB (L135) | C9 (macrocyclic core) | 3.9 |
|  | CD (K136) | C11 (macrocyclic core) | 3.7 |
|  | CZ (F154) | C7 (macrocyclic core) | 3.6 |
|  | CB (A157) | C12 (macrocyclic core) | 3.7 |
|  | SG (C159) | C13 (macrocyclic core) | 3.8 |

**References**

[1] Danelius, E., et al. MicroED as a powerful tool for structure determination of macrocyclic drug compounds directly from their powder formulations. *bioRxiv*, **2023**, doi: 10.1101/2023.07.31.551405.

[2] Unge, J., et al. Autonomous MicroED data collection enables compositional analysis. *ChemRxiv,* **2023**, doi: 10.26434/chemrxiv-2023-8qvwg.

[3] Kabsch, W. xds. *Acta Crystallographica Section D: Biological Crystallography*, **2010**, *66*(2), 125-132.

[4] Schneider, T. R., & Sheldrick, G. M. Substructure solution with SHELXD. *Acta Crystallographica Section D: Biological Crystallography*, **2002**, *58*(10), 1772-1779.

[5] Sheldrick, G. M. Crystal structure refinement with SHELXL. *Acta Crystallographica Section C: Structural Chemistry*, **2015**, *71*(1), 3-8.

[6] Pettersen, E. F., et al. UCSF ChimeraX: Structure visualization for researchers, educators, and developers. *Protein Science*, **2021**, *30*(1), 70-82.

[7] Macrae, C. F., et al. Mercury 4.0: From visualization to analysis, design and prediction. *Journal of applied crystallography*, **2020**, *53*(1), 226-235.

[8] Rossi Sebastiano, M., et al. Impact of dynamically exposed polarity on permeability and solubility of chameleonic drugs beyond the rule of 5. *Journal of Medicinal Chemistry*, **2018**, *61*(9), 4189-4202.

[9] Wieske, L. H., et al. Going Viral: An Investigation into the Chameleonic Behaviour of Antiviral Compounds. *Chemistry–A European Journal*, **2023**, *29*(8), e202202798.

[10] Cummings, M. D., et al. Induced‐fit binding of the macrocyclic noncovalent inhibitor TMC435 to its HCV NS3/NS4A protease target. *Angewandte Chemie International Edition*, **2010**, *49*(9), 1652-1655.

[11] Friesner, R. A., et al. Glide: a new approach for rapid, accurate docking and scoring. 1. Method and assessment of docking accuracy. *Journal of medicinal chemistry*, **2004**, *47*(7), 1739-1749.
